# Donated human milk use and subsequent feeding pattern in neonatal units

**DOI:** 10.1186/s13006-019-0233-x

**Published:** 2019-09-02

**Authors:** Wesam Alyahya, Debbie Barnett, Andrew Cooper, Ada L. Garcia, Christine A. Edwards, David Young, Judith H. Simpson

**Affiliations:** 1 Human Nutrition, School of Medicine, Dentistry and Nursing, College of Medical, Veterinary and Life Sciences, University of Glasgow, Glasgow, UK; 2 Neonatal Intensive Care Unit, Royal Hospital for Children, Glasgow, UK; 3 Department of Mathematics and Statistics, University of Strathclyde, Glasgow, UK

**Keywords:** Donated human milk, Milk bank, Human milk, Breastfeeding, Premature infants

## Abstract

**Background:**

Donated human milk (DHM) is a safe alternative in the absence of mother’s own milk (MOM); however, specific clinical indications for DHM use and its impact on subsequent feeding practice remain unclear. We aimed to audit local DHM use and explore the impact of the introduction of DHM as the first enteral feed on subsequent MOM availability.

**Methods:**

We retrospectively audited DHM recipients nursed in Royal Hospital for Children, Glasgow from 2014 to 2016 against local guidelines. Data were collected from an operational electronic database. Descriptive data analysis was performed to describe DHM use. To explore the association between the first human milk feed with subsequent MOM availability Kruskal Wallis test was used. Adjustments for confounding variables were performed using analysis of variance (ANOVA).

**Results:**

A total of 165 recipients of DHM (5.3% of all admission to RHC) were identified. The majority of recipients (69%) were born < 32 weeks of gestation. The main indication for DHM was prematurity, other indications included congenital anomalies of bowel and heart. The local guideline was adhered to in 87% of cases. The median interquartile range (IQR) at DHM introduction was 6 days (3, 17) and the duration of use was 12 days (6, 22). In those born < 32 weeks of gestation the type of human milk (DHM and/ or MOM) used as first feed did not influence the subsequent median IQR days of feeding with any MOM [DHM 40 (9, 51); MOM 28 (17, 49), MOM & DHM 17 (10, 26) *p* value = 0.465] after adjusting for birthweight and length of hospital stay.

**Conclusions:**

In our unit, DHM is mainly used in preterm neonates in accordance with existing local guidance. Using DHM as first milk feed did not affect subsequent MOM availability.

**Electronic supplementary material:**

The online version of this article (10.1186/s13006-019-0233-x) contains supplementary material, which is available to authorized users.

## Background

Mother’s own milk (MOM) is the optimal feed for all newborn babies, especially the sickest and most vulnerable. When insufficient MOM is available donated human milk (DHM) is recommended as an alternative [1–3]. DHM differs from MOM for a variety of reasons, many of which relate to the handling and processing of the milk. The nutritional content of DHM varies greatly, with a mean difference in energy intake of 38.7 kcal/kg/day based on full enteral feeds of 180 ml/kg/day [4, 5]. In addition, freezing, storage and heat treatment all impact on milk components and qualities. The Holder pasteurisation method (heating milk for 30 min at 62.5 °C) can reduce both fatty and amino acid content in DHM. For example, in one study the fatty acid content of DHM was 22% lower after pasteurisation [6]. Another study found the mean difference of valine (an amino acid) content in pasteurised DHM was lower by 22 mmol compared to EBM from healthy women [7]. Bioactive proteins such as immunoglobulins which are anti-inflammatory and important for immune modulation are lower in DHM than MOM because of pasteurisation [8]. Despite these differences, DHM has been demonstrated to retain some of the benefits of MOM, in particular, a reduction in the incidence of necrotising enterocolitis (NEC). A recent Cochrane meta-analysis of nine clinical trials (1017 preterm infants) showed that, compared with formula feeding, DHM was significantly associated with a lower risk of NEC [9, 10]. Given that approximately 50% of NEC cases require surgery or die [11], and that survivors are at risk of sequelae including; prolonged dependence on parental nutrition, short bowel syndrome and impaired neurodevelopment [12, 13], any intervention that reduces the risk of NEC is clinically and economically important. It is however well recognised that further research is required to inform practice and assist in prioritising DHM distribution [14].

A frequently cited concern associated with DHM use is a potential negative impact on the provision of MOM. A recent systematic review provided reassurance that breastfeeding rates are not adversely affected by the use of DHM, however, it highlighted the limited evidence base from which it drew its conclusions [15]. In particular, it is unclear whether using DHM as the first enteral feed will adversely influence subsequent MOM supply.

In the United Kingdom there are operational guidelines for milk banks but no national guidance on clinical indications for DHM usage [16]. In the absence of evidence based, cost effective eligibility criteria, local guidelines are often used to inform practice. The primary aim of this work was to audit the use of DHM in our neonatal unit, comparing it to local guidance. The secondary aim was to explore the impact of using DHM as the first milk on subsequent feeding practice.

## Methods

### Setting

The Scotland wide milk banking service, hosted by Greater Glasgow and Clyde Health Board, was officially launched in 2013 to provide DHM equitably across the country. Donated milk is processed and stored in Glasgow prior to transfer for use in neonatal units within the 14 Scottish health boards. The neonatal unit in the Royal Hospital for Children (RHC), Glasgow provides local perinatal care, regional neonatal surgical services and national neonatal cardiac and extracorporeal life support services. Due to the nature of these services, babies from across Scotland may be transferred into the RHC for specialist care at some point during their postnatal course.

### Data

Using the milk bank database all infants who received DHM over a three year period following the expansion of the milk banking service (January 2014 to December 2016) were identified. We retrospectively audited all recipients who were nursed in the RHC, Glasgow at any point during their postnatal stay (inborn and transferred in postnatally for specialist care). Caldicott Guardian approval, which is required for research involving patient data collection, was obtained.

Data were collected from an electronic medical record platform (Badger.net). Data collected included demographic characteristics (gestational age, birthweight and length of hospital stay), feeding history (age at initiation and type of first milk feed, age at initiation and indication for starting DHM, duration of feeding with DHM solely or mixed with other milk). We audited this against Greater Glasgow and Clyde local guidelines for DHM use which are; prematurity born < 32 weeks of gestational age, refeeding post NEC and congenital anomalies of the bowel and heart. The time of discontinuing DHM was based on clinical decision. NHS Greater Glasgow and Clyde local guidelines for DHM feeding are available in the Additional file 1.

### Analysis

Descriptive data analysis was performed using Microsoft Excel and IBM SPSS Statistics Data Editor, version 21. Tests included frequencies, median and interquartile range (IQR) as data were nonparametric. Comparison of all DHM users’ characteristics, feeding and length of stay was done using Kruskal Wallis test. Subgroup analysis for infants born < 32 weeks of gestation was performed also using the Kruskal Wallis test to explore the association between the first human milk feed with subsequent MOM availability (i.e. feeding with any MOM on its own or with other milk expressed in days of feeding and proportion of feeding days over the admission). Two babies born < 32 weeks of gestation who received formula for their first feed were excluded from this analysis. If the overall Kruskal Wallis test was significantly different (< 0.05), a post hoc test was done using pairwise comparison of independent samples (Kruskal Wallis 1-way ANOVA (K samples) to determine groups that differed from each other. Adjustments for confounding variables (birthweight and length of hospital stay) were performed using analysis of variance (ANOVA).

## Results

We identified 279 recipients of DHM across Scotland over the audit period; 88 in 2014 increasing to 108 in 2016. Of these, 165 (56%) were managed at some point in the RHC, Glasgow. This cohort represented 5.3% of all admissions to the RHC neonatal unit over the three-year audit period.

The majority of DHM recipients were preterm, 114 of whom were born < 32 weeks of gestation and 30 between 32 and 36^+ 6 days^ weeks of gestation. The remainder (21) were term babies who received DHM mainly because they had a congenital anomaly (Table 1). Other indications for using DHM for the group as a whole included congenital bowel disease (7%), congenital heart disease (7%), and refeeding following NEC (2%). Twenty-one infants (13%) received DHM differing from existing guidelines. The majority of babies (76%) received DHM to supplement MOM.

**Table 1:** Characteristics and feeding pattern of DHM recipients (*N* = 165)

	Very Preterm < 32 weeks	Late preterm 32–36^+ 6^ weeks	Term ≥ 37 weeks	*p* values
Number of babies (%)	114 (69%)	30 (18%)	21 (13%)	
Gestational age (weeks)	28 (26, 30)^a ,b^	33 (32, 35)^a^	38 (37, 39)^b^	< 0.001
Birthweight (grams)	1040 (835, 1246)^a, b^	1893 (1459, 2195)^a^	2910 (2410, 3442)^b^	< 0.001
Feeding initiation age (days)	3 (2, 4)	2 (2, 4)	5 (2, 10)	0.916
DHM feeding				
Initiation age (days)	7 (3, 19)	5 (3, 8)	5 (4, 14)	0.211
Any DHM duration (days)	14 (6, 25)^a^	10 (5, 14)^a^	9 (4, 15)	0.001
Length of stay (days)	66 (54, 108)^a, b^	28 (21, 50)^a^	28 (9, 65)^b^	< 0.001
DHM indication (number)				
Prematurity	114	23		
Congenital heart disease	1	4	6	
Congenital bowel anomaly		2	9	
Other	1	1	6	

Data presented as median (interquartile range), *MOM* mother’s own milk, *DHM* donated human milk, Length of stay represent infants’ hospital admission including their stay in the Royal Hospital for Children, *p* value was calculated based on Kruskal Wallis test, Post hoc analysis was done using pairwise comparison of independent samples, Kruskal Wallis 1-way ANOVA (K samples). Superscripts are significantly different for comparisons between groups (^a^very preterm versus late preterm, ^b^very preterm versus term, ^c^late preterm versus term)

**Fig. 1:**
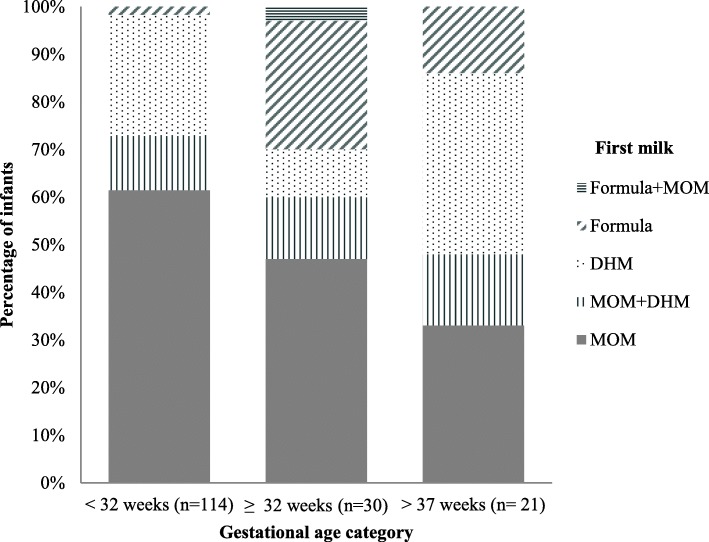
Proportion of babies according to the type of first milk of feeding in donated human milk recipients (*n* = 165). Footnotes: *MOM:* mother’s own milk, *DHM:* donated human milk

The median (IQR) age at initiation of feeds for the group as a whole was three days (2, 4). The age at initiation in differing gestational age groups is shown in Table 1. Two-thirds of those born < 32 weeks of gestation received MOM as their first feed compared to a third of term babies. Only two babies born < 32 weeks of gestation received formula rather than human milk (MOM or donated) as their first feed. The highest proportion fed DHM as first milk was in term infants (Fig. 1).

The median (IQR) age at introduction of DHM for the group as a whole was six days (3, 17), and the median duration of use was 12 days (6, 22). The age at introduction and duration of DHM use in differing gestational age groups is shown in Table 1. Those born < 32 weeks of gestation received DHM for longer than the more mature groups (Table 1).

**Fig. 2:**
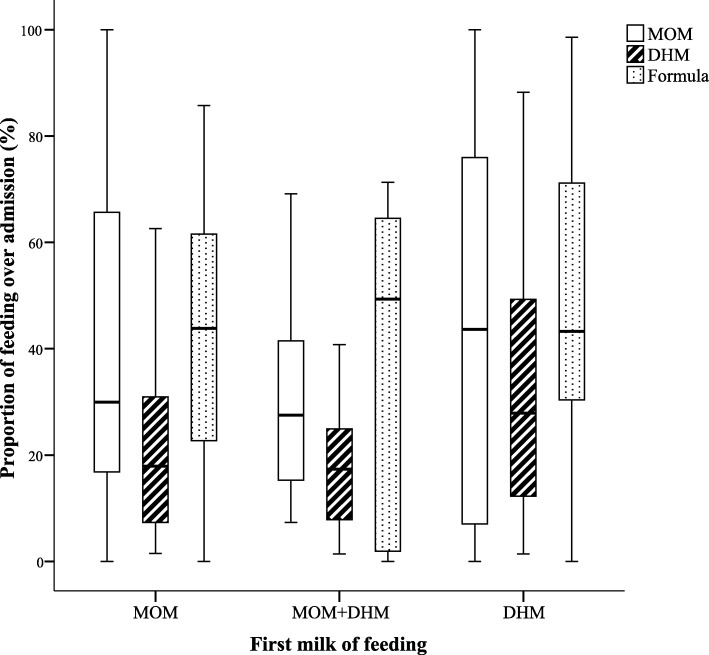
Feeding pattern over admission in infants born < 32 weeks of gestation (*n* = 112). Footnotes: Box plots represent the median (interquartile range) of feeding pattern over admission expressed as percentage of days over hospital stay. *MOM:* mother’s own milk, *DHM:* donated human milk

Subsequent milk feeding in babies born < 32 weeks of gestation who received human milk (MOM and/or DHM) as their first feed is described in Fig. 2. There was no significant difference in the number of days of feeding with any MOM (after correction for birthweight and length of hospital stay) and proportion of any MOM intake over the admission period based on the first milk of feeding (Table 2).

**Table 2:** Milk feeding according to the first human milk fed in infants born < 32 weeks of gestation*

	First milk of feeding			
	MOM	MOM & DHM	DHM	*p* value
Number of babies	70	13	29	
Gestational age (weeks)	27 (25, 29) ^b^	29 (27, 30)	28 (27, 30)^b^	0.009
Birth weight (grams)	988 (780, 1175) ^b^	1262 (980, 1360)	1140 (920, 1440)^b^	0.015
Length of stay (days)	94 (63, 120)^a, b^	71 (41, 82)^a, b^	69 (37, 94)	0.002
Age of feeding initiation	3 (2, 4)	3 (2, 4)	3 (2, 4)	0.494
Human milk feeding				
Any MOM (days)	28 (17, 49)	17 (10, 26)	40 (9, 51)	0.465^1^
Proportion over admission (%)	29 (16, 65)	28 (15, 55)	44 (5100)	0.135

Data presented as median (interquartile range), *MOM* mothers’ own milk, *DHM* donated human milk, Length of stay represent infants’ hospital admission including their stay in the Royal Hospital for Children, *p* value was calculated based on Kruskal Wallis test, Post hoc analysis < 0.05 was done using pairwise comparison of independent samples, Kruskal Wallis 1-way ANOVA (K samples)^1^ *p* value for any MOM was adjusted for birth weight and length of hospital stay. Superscripts are significantly different for comparisons between groups (^a^MOM versus MOM & DHM, ^b^MOM versus DHM, ^c^MOM & DHM versus DHM)

^*^Two babies fed formula as first milk were excluded from this analysis

## Discussion

Our data provide reassurance that DHM use in RHC adheres to the current guidance in the majority of cases. The commonest deviation from this guidance is in the late preterm group (32–36^+ 6^ weeks). This may be due to the lack of a clear evidence base to guide clinical use [3, 17], it may also reflect therapeutic creep or parental request as awareness of, and access to DHM increases [18, 19]. It is encouraging to see that the majority of babies (76%) received DHM as a supplement to, rather than in place of MOM and this replicates the finding of others throughout the United Kingdom [20, 21] and the United States [18]. The median age at initiation of enteral feeds for the group as a whole was three days, in part reflecting the time it can take to establish lactation following the delivery of a preterm and/or sick baby.

Access to DHM has been associated with positive in-hospital feeding outcomes, such as earlier initiation of feeds, faster feed advancement and increased volumes of MOM [22, 23] but equally early (< 48 h) introduction of DHM has been linked to reduced MOM availability [24]. In our population of babies born < 32 weeks of gestation the type of first human milk (MOM or donated) did not appear to have a negative impact on subsequent MOM availability. This raises the possibility of introducing enteral feeds of DHM at an earlier stage with a view to expediting the time to establish full milk feeds and minimising the duration (and risks) of central venous access and parenteral nutrition [25]. Ideally, any such change in practice should be subject to randomised evaluation or at the very least quality improvement methodology to ensure that it did not adversely impact maternal milk provision or mitigate the benefits of early colostrum administration.

In our sample, over 60% of babies born < 32 weeks of gestation received MOM as first feed compared to only a third of term babies (the majority of whom had congenital anomalies of either bowel or heart, and around half of whom did not have any MOM during their hospital stay). The two groups are obviously very different, but this difference in access to MOM may reflect the emphasis placed on supporting early milk expression for the preterm population. It is important to remember that MOM confers many benefits at any gestation age, especially in those vulnerable infants requiring admission to a neonatal unit. DHM use in term infants appears to be increasing. A recent American study described the use of DHM in healthy term infants to reduce exposure to formula milk whilst lactation was established. They reported higher exclusive breastfeeding at discharge [26]. However, a recent randomised clinical trial found early DHM supplementation in term infants did not significantly increase breastfeeding at the age of one week or three months [27]. Although DHM feeding in term newborns may reduce formula exposure, the effect on breastfeeding duration and formula exposure needs further investigation. Given the public health implications of improved societal breastfeeding rates the potential role of DHM in this situation is worthy of further study. The Scottish Government has recently funded a quality improvement initiative whereby DHM will be used to support breastfeeding mothers on the postnatal wards if the baby requires a supplementary feed. The primary aim is to facilitate the establishment and maintenance of breastfeeding by avoiding the negative impact of early formula feeding [28].

## Conclusion

Whilst uncertainty around the optimal clinical indications for DHM remain, it is important that its use is monitored. Our data suggested that in Scotland adherence to current recommendations is good and that judicious DHM use in the preterm population is not negatively impacting maternal milk availability. However, it is clear that further study is required to fully delineate the role of DHM in contemporary neonatal care.

## Additional file

Additional file 1: Criteria for who should be offered donated human milk (based on NHS Greater Glasgow and Clyde guidelines). (DOC 23 kb)

## Data Availability

The dataset analysed in this study is available from Prof Edwards on reasonable request.

## Abbreviations

DHM: Donated human milk
MOM: Mother’s own milk
NEC: Necrotising enterocolitis
RHC: Royal Hospital for Children
