# Supplementary material for: Donated human milk use and subsequent feeding pattern in neonatal units
Source: Int Breastfeed J. 2019 Sep 2;14:39. doi: 10.1186/s13006-019-0233-x (PMC6721171; doi:10.1186/s13006-019-0233-x)
Supplement: Supplementary file 1 — Criteria for who should be offered donated human milk (based on NHS Greater Glasgow and Clyde guidelines). (DOC 23 kb) [file 13006_2019_233_MOESM1_ESM.doc]

**Additional file**

**Criteria for who should be offered donated human milk (based on NHS Greater Glasgow and Clyde guidelines)**

The purpose is to establish milk feeds in high risk infants when MEBM is unavailable or insufficient. This is to provide short term support whilst a mother is establishing milk expression.

The indications include:

- Infants <32 weeks gestation
- Infants whose birth weight <1500 grams
- Previous proven NEC ± laparotomy
- Post GI surgery for congenital bowel anomaly
- Congenital heart disease with potential for gut hypo perfusion

This list is not exhaustive and additional provision is decided on a case by case basis.
